# Marijuana Use in Adults Living with Sickle Cell Disease

**DOI:** 10.1089/can.2018.0001

**Published:** 2018-07-01

**Authors:** John D. Roberts, Jonathan Spodick, Joanna Cole, Janis Bozzo, Susanna Curtis, Ariadna Forray

**Affiliations:** ^1^ Yale Cancer Center, Yale University, New Haven, Connecticut.; ^2^ Department of Social Work, Yale New Haven Hospital, New Haven, Connecticut.; ^3^ Department of Community Health Outpatient Practice, Yale New Haven Hospital, New Haven, Connecticut.; ^4^ Department of Information Technology Services Analytics Strategy, Yale New Haven Health, New Haven, Connecticut.; ^5^ Department of Psychiatry, Yale University, New Haven, Connecticut.

**Keywords:** marijuana, medical marijuana, sickle cell disease, cannabinoid

## Abstract

**Introduction:** Legal access to marijuana, most frequently as “medical marijuana,” is becoming more common in the United States, but most states do not specify sickle cell disease as a qualifying condition. We were aware that some of our patients living with sickle cell disease used illicit marijuana, and we sought more information about this.

**Materials and Methods:** We practice at an urban, academic medical center and provide primary, secondary, and tertiary care for ∼130 adults living with sickle cell disease. We surveyed our patients with a brief, anonymous, paper-and-pen instrument. We reviewed institutional records for clinically driven urine drug testing. We tracked patient requests for certification for medical marijuana.

**Results:** Among 58 patients surveyed, 42% reported marijuana use within the past 2 years. Among users, most endorsed five medicinal indications; a minority reported recreational use. Among 57 patients who had at least one urine drug test, 18% tested positive for cannabinoids only, 12% tested positive for cocaine and/or phencyclidine only, and 5% tested positive for both cannabinoids and cocaine/phencyclidine. Subsequent to these studies, sickle cell disease became a qualifying condition for medical marijuana in our state. In the interval ∼1.5 years, 44 patients have requested certification.

**Conclusion:** Our findings and those of others create a rationale for research into the possible therapeutic effects of marijuana or cannabinoids, the presumed active constituents of marijuana, in sickle cell disease. Explicit inclusion of sickle cell disease as a qualifying condition for medical marijuana might reduce illicit marijuana use and related risks and costs to both persons living with sickle cell disease and society.

## Introduction

In the past 20 years, 29 states and the District of Columbia have enacted laws permitting the legal use of marijuana for medicinal purposes.^1^ A common rationale is that marijuana has medicinal properties, including relief of pain.^2,3^ Although some laws include very general qualifying conditions, such as “chronic pain,” all contain lists of specific qualifying medical diagnoses, including conditions that may involve chronic pain such as “cancer” or “multiple sclerosis.” Only three states (Connecticut, Ohio, and Pennsylvania) specifically list sickle cell disease as a qualifying condition for medical marijuana.^1^

The Committee on Health Effects of Marijuana of the National Academies of Sciences, Engineering, and Medicine has found that there is conclusive or substantial evidence that cannabis (marijuana) or cannabinoids (natural marijuana constituents or their synthetic analogs) are effective for the treatment of chronic pain in adults.^4^ Although the report mentions scores of diseases, there is no mention of sickle cell disease.

Pain, both acute and chronic, is the hallmark of sickle cell disease.^5,6^ Pain in sickle cell disease is unlike other chronic painful conditions; however, in sickle cell disease chronic pain generally has been preceded by years of episodes of acute pain that result in acute health services utilization and administration of parenteral opioids, and these episodes continue after the onset of chronic pain.^5,6^

We were aware that some of our patients used marijuana, often for the stated reason of pain relief. Of interest, a cannabinoid has been shown to have antinociceptive effects in a transgenic mouse model of sickle cell disease.^7,8^ We were also aware that a few of our patients had been incarcerated for marijuana-related activities. But little has been reported about marijuana use in persons living with sickle cell disease. To better understand marijuana use among our patients, we reviewed findings from urine drug testing done as part of our routine clinical care; we surveyed a sample of our patients about marijuana use; and we tracked requests for certification among our patients after the addition of sickle cell disease to the list of qualifying conditions for medical marijuana.

## Materials and Methods

We practice in an urban, academic medical center that provides primary, secondary, and tertiary care for persons living with sickle cell disease. Almost all scheduled outpatient primary and hematological care for adults (persons 21 years and older) is provided through our clinic. At the time of these studies, ∼130 patients were engaged in our program, defined as two visits to the outpatient sickle cell clinic within an 18-month period. Our patients have a median age of 31 years, and ∼60% are women. In our clinic, the mainstay of treatment for both acute and chronic pain is opioids. We make no issue about marijuana use, but evidence of use of other illicit drugs, such as cocaine, phencyclidine, or nonprescribed opioids, results in sanctions such as temporary cessation of opioid prescribing.

We have a policy of obtaining urine drug tests at least once yearly in all patients receiving prescriptions for significant amounts of opioids and more frequently in selected patients, especially patients about whom we have concerns about inappropriate use of opioid medications or other drugs on the basis of patient report, behavior, or urine drug test results. We routinely order a panel that tests for cannabinoids, cocaine, phencyclidine, as well as amphetamines, barbituates, and a variety of opioids. Some of our patients also undergo testing ordered by other clinicians, especially in the emergency department. From hospital laboratory data, we identified all urine drug tests obtained from our patients from February 1, 2013 through August 30, 2014. As it would have been difficult to distinguish licit from illicit use of amphetamines, barbituates, and various opioids, which could have been prescribed in the past or by others, this analysis focused upon results for cannabinoids, cocaine, and phencyclidine, all of which represented illicit drug use.

We developed and validated by repeated testing in surrogate patients a self-report survey about marijuana use (Supplementary Data). In 2015, we invited patients seen in clinic in the months of March, April, and May to complete this survey in an anonymous manner. At the time, our state (Connecticut) had a medical marijuana program; but sickle cell disease was not a qualifying condition, and we were not aware of any patient who had been certified for medical marijuana on any other basis. Thus, all reported use was presumably illicit. We solicited self-assigned gender information, but, to avoid the appearance of collected data that could be used to deanonymize results, we did not solicit age. A verbal consent was obtained by a clinic doctor (J.D.R.) or nurse practitioner (J.C.).

We had a number of implicit hypotheses about marijuana use that can be inferred from the survey questions (Supplementary Data), but we stated no *a priori* hypotheses and planned no formal testing for significance of differences.

The marijuana survey and urine drug testing studies were conducted as approved by the Yale University institutional review board. Other data were collected as part of ongoing monitoring of our adult sickle cell program.

## Results

Fifty-seven patients, 28 men and 29 women, ∼44% of our patient population, had undergone urine drug testing. Among patients tested, the median number of tests was 2 (range 1–21). Ten patients (18%) had positive tests for cannabinoids only; three (5%) had positive tests for both cannabinoids and cocaine/phencyclidine; and seven (12%) had positive tests for cocaine and/or phencyclidine only. Among patients testing positive for cannabinoids, six were men and seven were women.

All 58 invited patients, 20 men and 38 women, ∼45% of our patient population, agreed to participate in the survey. We observed no reluctance to participate and heard no comments to suggest that illicit marijuana use was a stigmatized behavior among our patients or their family members or friends attending clinic. Forty-two percent of patients reported use of marijuana within the past 2 years (Table 1). Reported frequency of use was approximately evenly distributed among four frequency categories: less than monthly, monthly, weekly, and daily. Among users, a majority endorsed all five survey-listed medicinal reasons for marijuana use (pain, anxiety, mood, sleep, and appetite); one-third reported using marijuana to get high. Seventy-nine percent reported that marijuana allowed less use of pain medicine. Two-thirds of men and one-third of women reported marijuana use (data not shown). Among users, men reported somewhat more frequent use (data not shown).

**Table 1.** **Self-Report of Marijuana Use in Adults Living with Sickle Cell Disease (58 Subjects)**

Use within 2 years	Yes					
	42%					
Frequency	<Monthly	Monthly	Weekly	Daily		
	21%	13%	29%	33%		
Rationale	Pain	High	Anxiety	Mood	Sleep	Appetite
	92%	33%	71%	67%	71%	63%
Reduces use of pain medications	Yes					
	79%					

In March 2016, sickle cell disease became a qualifying condition for medical marijuana in our state. In the absence of any relevant studies on sickle cell disease, we never recommend marijuana, medicinal or otherwise, to our patients. We do inform patients of their eligibility for medical marijuana, and especially for those who use illicit marijuana, we point out that use of medical marijuana reduces the risk of inadvertent toxic exposures and criminal sanctions. In the ∼1.5 years since sickle cell disease became a qualifying condition for medical marijuana, 44 patients have requested certification; of these, we have certified 42 and refused to certify 2 due to our concerns about potential drug diversion.

## Discussion

In our urban academic medical center clinic for adults living with sickle cell disease, 23% of 57 patients who underwent urine drug testing in an 18-month period tested positive for cannabinoids on at least one occasion. Somewhat fewer patients (18%) tested positive for cocaine and/or phencyclidine, and very few (5%) tested positive for both. In an anonymous survey, 42% of 58 patients surveyed reported marijuana use within the past 2 years. Among these, majorities endorsed use for medicinal purposes related to pain, anxiety, mood, sleep, and appetite, and a minority reported recreational use. A large majority reported that marijuana use allowed less use of pain medications. Subsequent to the addition of sickle cell disease to the list of qualifying conditions in our state, about one-third of our patients, including some nonmarijuana users, have requested certification for medical marijuana.

We are aware of only three other studies of marijuana use in adults with sickle cell disease. In 2005, Howard et al.^9^ reported that 36% of British adults living with sickle cell disease reported “ever use.” A vast majority endorsed marijuana use for what we have broadly classified as medicinal purposes, whereas 16% reported use “to get high.” In 2006, Knight-Madden et al.^10^ described self-reported marijuana use by ∼65% of men and ∼20% of women living with sickle cell disease in Jamaica. In 2017, Ballas^11^ reported findings from 15 years of random urine testing in adult patients living with sickle cell disease in the Philadelphia area. Ten percent of the patient population had ever been tested with a mean of ∼4 tests each. Of these patients, ∼50% tested positive for cannabinoids on at least one occasion. Thus, multiple reports suggest that a substantial minority of adults living with sickle cell disease in the developed world use marijuana.

The 2013 U.S. National Survey on Drug Use and Health found that 19% of adults aged 18–25 years and 7% of adults aged 26 years and older reported using marijuana in the past month.^12^ There was no difference in use among races/ethnicities. Among our patients undergoing urine drug testing, 23% tested positive for cannabinoids on at least one occasion. This is likely an underestimate of use among these patients as urine would not be expected to be positive for cannabinoids for more than a few days in occasional users.^13^ In our survey, 31% of patients reported using marijuana within the past month. Thus, in comparison with the National Survey, both our urine testing results and survey results suggest that marijuana use is more common among adults living with sickle cell disease than in the general population.

Our patients reported that marijuana use allowed for less use of pain medications. As our survey was anonymous, we cannot independently validate their self-assessment. It is consistent with other survey data from persons with diverse types of chronic pain using medicinal marijuana.^14^ It is also consistent with changes in Medicare claims data for prescriptions of pain medications after enactment of a state medical marijuana law.^15^ Further, it is consistent with observations that the availability of medical marijuana is associated with fewer deaths and other problems related to opioids in the population at large.^16,17^

The high rate of requests for certification for medical marijuana, including requests from nonmarijuana users, suggests that our patients are interested in marijuana as a potential symptomatic treatment for the sickle cell disease.

Our study has limitations. Our use of urine testing results to estimate frequency of marijuana use had conflicting biases: infrequent testing would miss occasional users; but to the extent that testing was driven by clinician's concerns, patients more likely to use might have been more likely to be tested. In our survey, as patients were reporting on an illicit activity, it is possible that some users did not acknowledge marijuana use, and that acknowledging users exaggerated medicinal benefits and underreported recreational motivations. We surveyed patients attending clinic during a 3-month period. As patients with greater disease severity are likely to have more frequent visits, our survey sample likely was biased toward patients with greater disease severity, although whether such a bias would impact our findings is unknown. We have not yet gathered outcomes data following certification of patients for medical marijuana.

## Conclusion

Marijuana and its constituents have been shown to be effective for the treatment of diverse types of chronic pain in adults, but effects of marijuana have never been studied in persons living with sickle cell disease. From our study as well as a few other reports, it appears that many adults with sickle cell disease use marijuana in the belief that it has medicinal benefits. And, in a murine model of sickle cell disease, a cannabinoid had antinociceptive effects. Thus, there is a strong rationale for the study of the medicinal properties of marijuana and/or its constituents in sickle cell disease. In the interim, use of illicit marijuana places persons living with sickle cell disease at risk for both exposure to poisonous contaminants and arrest and incarceration. More widespread inclusion of sickle cell disease as a qualifying condition for medical marijuana might reduce the personal and social costs of illicit marijuana use.

## Supplementary Material

Supplemental data
