# Supplementary material for: Marijuana Use in Adults Living with Sickle Cell Disease
Source: Cannabis Cannabinoid Res. 2018 Jul 1;3(1):162–5. doi: 10.1089/can.2018.0001 (PMC6044416; doi:10.1089/can.2018.0001)
Supplement: Supplemental data [file Supp_Data.pdf]

Supplementary Data

This Survey is Anonymous and Confidential

Connecticut is considering adding sickle cell disease to the list of conditions that qualify for medical marijuana. We are interested in learning about marijuana use by people with sickle cell disease. You do not have to complete this form. Whether you complete the form will not affect your care.

Have you filled out this form before? ☐ Yes ☐ No

Have you used marijuana in the past 2 years? ☐ Yes ☐ No

If you answered yes,

Why do you use marijuana? Check yes as many times you like.

To relieve pain:	☐ Yes	☐ No
To get high:	☐ Yes	☐ No
To relieve anxiety:	☐ Yes	☐ No
To improve my mood:	☐ Yes	☐ No
To help with sleep:	☐ Yes	☐ No
To increase appetite:	☐ Yes	☐ No

How often do you currently use marijuana? Check one answer that is the closest.

☐ less than once a month
☐ every month
☐ every week
☐ every day

Does using marijuana allow you to use less of your pain medicines?

☐ Yes ☐ No

You are a ☐ Man ☐ Woman

Version 1-14 -15
